# Medical Laws of Alabama

**Published:** 1881-02

**Authors:** 


### CORRESPONDENCE.

### MEDICAL LAWS OF ALABAMA.

Messrs. Editors.—In the December number of your valuable journal, you commended with much earnestness, the action of the North Carolina Legislature in creating a State Medical Examining Board. Feeling assured that you are unacquainted with the work our State Association has accomplished, and that you are not aware of the almost perfect system it has adopted for the elevation of the standard of our noble calling; I deem it but just that the world be made acquainted with this system.

First, there is a State Medical Examining Board which holds an annual meeting during the session of our State Association. Its meetings are not limited to this one sitting, but, "called meetings" may be held as often as necessary. Subsidiary to the State Board, there is, in each county, a County Medical Examining Board.

Candidates for the practice of Medicine in this State, *whether they have a diploma or not, and it matters not from what College their diploma is held*, are compelled by the laws of the State to submit to an examination before either the State Board, or that of some county. Should either Board grant them a certificate to practice, they are required to have this certificate registered with the Probate Judge of the county in which they propose to carry on their profession. The County Boards are required to keep a record of the examination of every candidate, (this record includes all *questions* asked by the Board) and submit it to the State Board at its next annual meeting. This prevents any "loose" examinations by the County Boards. A heavy fine is the penalty attached for the attempt to practice Medicine after failure before an Examining Board.

Let me assure you, Messrs. Editors, that these are not *mere forms* to frighten the would-be practitioners. Numbers of men who go before these Boards feeling well posted are sent away empty handed. At the meeting of the association last April in Huntsville, there were *ten* applicants for diplomas from our State Board, to *only one of whom a diploma was granted.* This law applies equally to Physicians, Druggists, and Dentists, though I believe it has not been so rigidly enforced with the two latter.

Here, I am happy to note another "step in the right direction." "Until 1883, it is left to the discretion of the several County Societies to enforce, or not to enforce the rule in relation to the *preliminary* examination of persons desiring to begin the study of Medicine." This sentence taken from the Medical Transactions of the State of Alabama for 1880, sufficiently explains itself. A large number of the County Societies have already enforced this rule.

Thus, Messrs. Editors, you see that "the Profession" in Alabama is not lying dormant, but is ever vigilant, trying with all its energies to elevate the standard of our glorious profession, and lift it from the mire to which it has been dragged by unqualified and unprincipled members. I am proud to say, and feel sure that I am doing right in saying, that the Physicians of this State are better organized, and are doing more to advance the Standard of Medical Education, than the Physicians of any other State in the Union.

Orrville, Alabama, Jan. 30th, 1881. SUBSCRIBER.

